# Epiretinal membrane: an overview and update

**DOI:** 10.1007/s10384-024-01127-6

**Published:** 2024-10-28

**Authors:** Ryo Matoba, Yuki Morizane

**Affiliations:** Department of Ophthalmology, Graduate School of Medicine, Dentistry and Pharmaceutical Sciences, Okayama University, 2-5-1 Shikata-cho Kita-ku, Okayama City, Okayama 700-8558 Japan

**Keywords:** En face imaging, Epiretinal membrane, Epiretinal proliferation, Internal limiting membrane, Lamellar macular hole

## Abstract

Epiretinal membrane (ERM) is a frequently diagnosed macular disease associated with aging, characterized by a fibrous membrane forming on the internal limiting membrane (ILM) and leading to visual dysfunctions such as metamorphopsia. Various hypotheses regarding the pathology of metamorphopsia have been proposed; however, the complete pathophysiologic mechanism underlying ERM remains unclear. Optical coherence tomography (OCT) provides detailed images enabling precise diagnosis and characterization of ERM, with several recent studies using the latest OCT imaging techniques. Surgical removal of ERM is the only treatment option; however, criteria for surgical intervention are not established, complicating the decision-making processes. Furthermore, the debate on whether simultaneous peeling of the ILM during ERM surgery enhances outcomes or poses unnecessary risks is ongoing, with no definite conclusion having yet been reached. This review also focuses on epiretinal proliferation, which is different from ERM and is characteristic of lamellar macular hole (LMH). Recently, diagnostic criteria for LMH and related diseases were proposed. Reports on effective surgical procedures for LMH exist, although more research is needed to confirm the long-term outcomes. Thus, this review article aims to provide an overview and updated knowledge of ERM, LMH, and related diseases.

## Introduction

Epiretinal membrane (ERM) is a common macular disease encountered in daily clinical practice. Epidemiologic studies have shown that that the prevalence of ERM ranges from 4 to 11.8% and that ERM is associated with aging [1–4]. A translucent fibrous membrane forms on the internal limiting membrane (ILM) of the macula. This membrane can tractionally distort the retina, causing morphologic abnormalities and visual dysfunctions such as metamorphopsia, decreased visual acuity, and macropsia [5–8]. ERMs are often formed as a result of aging and are termed idiopathic when no specific cause is identified. Conversely, secondary ERMs may develop following diseases such as diabetic retinopathy, uveitis, retinal tears, retinal detachment, retinal vascular occlusions, and retinitis pigmentosa [9–15]. ERM is diagnosed using ophthalmoscopic findings and optical coherence tomography (OCT), and the only treatment, regardless of the cause, is the surgical removal of the ERM [16]. Thus, ERM is a frequently diagnosed condition with relatively well-established protocols for diagnosis and treatment. However, unresolved challenges, such as an incomplete understanding of the ERM pathophysiology and undefined criteria for surgical intervention, persist. In this review, we aim to discuss the pathophysiology and treatment of ERM and lamellar macula hole (LMH), a related disease to ERM, on the basis of the latest findings from recent imaging studies.

### Pathophysiology

The primary cells that comprise the ERM are retinal glial cells such as Müller cells, hyalocytes, retinal pigment epithelial cells, and myofibroblasts [17–19]. Although the pathogenesis of idiopathic ERM is not fully elucidated, 2 main hypotheses have been proposed. One is that retinal glial cells, such as Müller cells, migrate onto the retinal surface. This migration is triggered by the cleavage in the ILM caused by strong traction on the area of strong adhesion between the vitreous and retina during posterior vitreous detachment [20]. The second theory is that the residual posterior vitreous cortex is the origin of ERM formation. Owing to vitreoschisis and vitreoretinal traction caused by anomalous posterior vitreous detachment, the vitreous cortex remains on the macula. Hyalocytes in the vitreous remnants are stimulated by various cytokines, such as basic fibroblast growth factor and nerve growth factor, and proliferate and differentiate into myofibroblasts, leading to ERM formation [21–24].

In both mechanisms, the production of various growth factors and cytokines is increased, and Müller cells and hyalocytes are transformed into myofibroblasts (an important molecular biologic mechanism in intraocular proliferative diseases, including ERM) [25–27]. Consequently, extracellular matrices such as collagen and contractile proteins such as alpha-smooth muscle actin are produced, forming pathologic ERMs with contractile properties.

### Diagnosis and imaging analyses

ERM is diagnosed by use of ophthalmoscopy. Typical findings include an irregular reflex in the macular region and retinal folds caused by ERM traction. To diagnose secondary ERM, a detailed history and observation of ocular findings related to secondary ERM, such as peripheral retinal tears and ocular inflammation, is important.

**Fig. 1:**
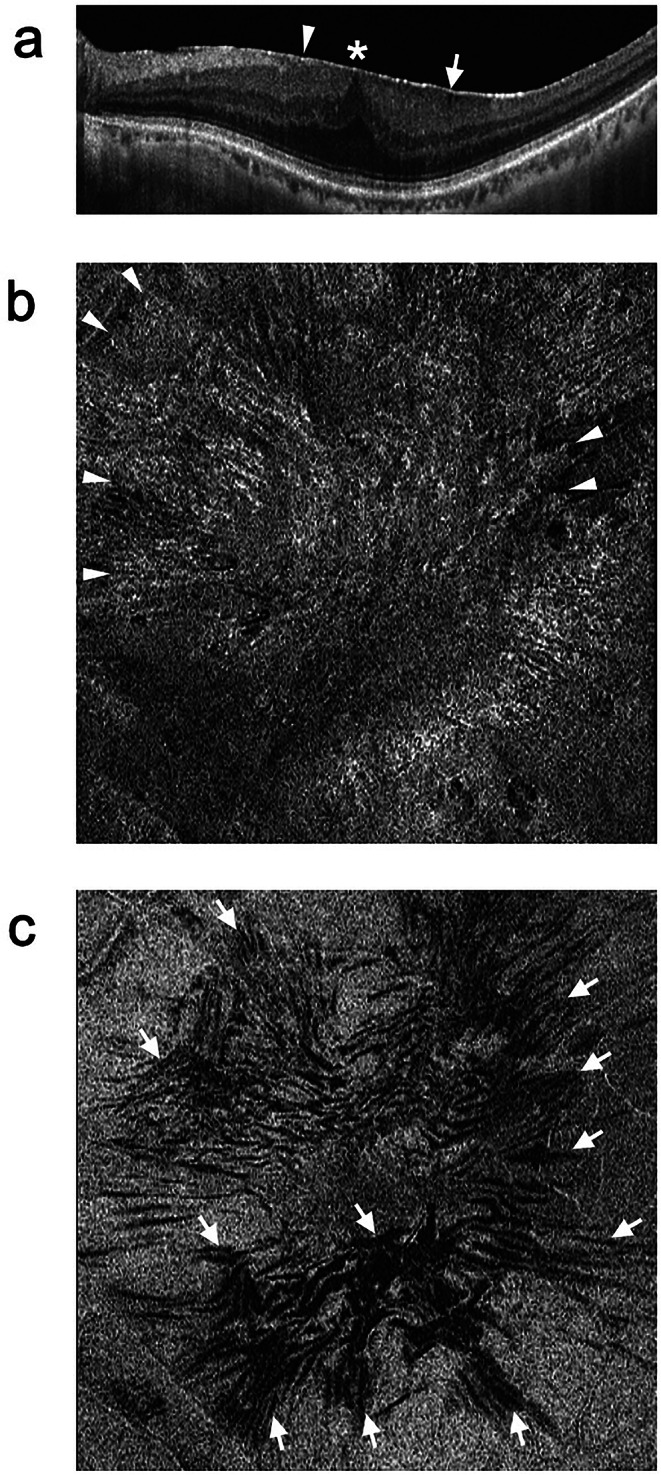
Representative optical coherence tomography (OCT) image of epiretinal membrane (ERM). Horizontal B-scan image **(a)** and en face image flattened along the internal limiting membrane (ILM) (**b** and **C**). **a** ERM is observed as a hyperreflective line on the retinal surface (arrowhead). A retinal fold (arrow) is also seen. Owing to the retinal traction caused by ERM, the foveal pit has disappeared, and ectopic inner foveal layers exist (asterisk). **b** En face image at the ILM level shows the ERM as a hyperreflective membranous lesion (arrowheads). **c** En face image 30 μm below the ILM level shows the retinal folds as hyporeflective linear lesions (arrows)

OCT is the most useful tool for ERM diagnosis. On B-scan images, ERMs are observed as hyperreflective linear structures on the retinal surface (Fig. 1a). Retinal folds are often observed as localized depressions on the retinal surface owing to the retinal traction by the ERM. In advanced ERM, the foveal pit disappears, and the inner layers of the retina are seen over the fovea where they do not normally exist (ectopic inner foveal layers) (Fig. 1a) [28]. Additionally, abnormal findings such as cotton-ball signs and foveolar detachment may be present in the outer retina [29, 30]. Thus, B-scan images are useful for evaluating changes in the retinal layer structures due to ERM. Moreover, en face images are beneficial, as they are constructed from 3-dimensional images of the retina and can provide a bird’s eye view of the structure in any layer of the retina. For example, on the en face images flattened along the ILM, the ERM is observed as a hyperreflective membranous lesion at the ILM level (Fig. 1b), and the retinal folds are observed as hyporeflective linear lesions below the ILM level (Fig. 1c) [31]. En face images can accurately detect even subtle ERM by allowing evaluation of the entire macula and can visualize the degree of retinal deformation due to ERM by observation of the retinal folds [7, 8, 13, 31–34]. Evaluating ERM from multiple perspectives utilizing the advantages of the B-scan and en face images as described above is important.

### Pathology of visual function disturbance

Previous studies have proposed 3 main hypotheses as mechanisms for how ERM causes visual function disturbance: (1) photoreceptor abnormalities, (2) inner retinal layer damage, and (3) Müller cell displacement. The first hypothesis suggests that visual dysfunction is caused by the traction of photoreceptor cells by Müller cells. OCT images of ERM occasionally show abnormalities in the outer retinal layers [29, 30], and “microfolds” are observed in the photoreceptor layer in adaptive optics scanning laser ophthalmoscopy [35], suggesting photoreceptor displacement. Additionally, photoreceptor cells are less sensitive to light from oblique angles (Stiles–Crawford effect) and may be involved in visual impairment [36–38]. The second hypothesis is that metamorphopsia is caused by damage to the inner nuclear layer (INL) (including Müller, horizontal, bipolar, and amacrine cells) owing to the retinal traction caused by ERM. The decrease in b-waves and oscillatory potential amplitudes in focal macular electroretinograms [8, 36] and the significant correlation between the degree of metamorphopsia and the INL thickness [5, 39] support this theory. The third hypothesis focuses on the light-guiding function of Müller cells. Müller cells have a funnel-shaped structure on the ILM side and roughly one-to-one contact with cone cells on the photoreceptor side in the fovea [40]. Müller cells act like optical fibers that collect light projected into the retina and transmit it to the cone cells or surrounding rod cells because of their unique structure. Therefore, retinal traction caused by ERM might displace Müller cells toward the fovea. Moreover, the light is transmitted from Müller cells, which are not normally involved in light collection, to the corresponding photoreceptor cells, resulting in metamorphopsia and macropsia [41].

### ERM treatment

**Fig. 2:**
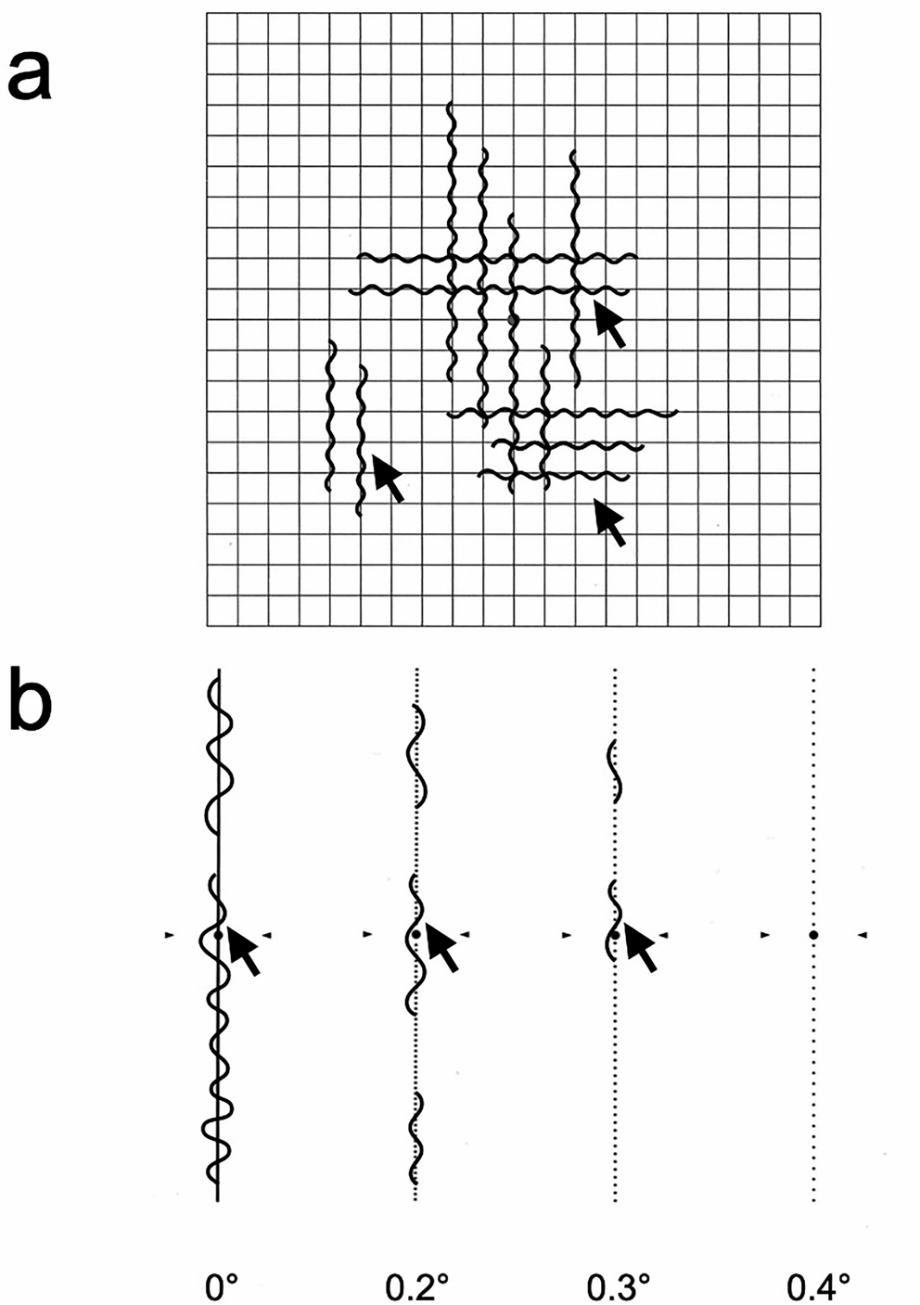
Schematic image showing 2 types of metamorphopsia test. **a** Amsler chart. The patient is asked to stare at the center of a 10-cm-square chart with grid lines and indicate the distortion or waviness of the lines. In this case, the patient has metamorphopsia (arrows), although its severity is unknown. **b** M-CHARTS (Inami). This is composed of inspection sheets with a straight line and 19 dotted lines with dot intervals ranging from 0.2 to 2.0 degrees of visual angle. In this case, the patient has metamorphopsia (arrows) at 0 to 0.3 degrees and perceives the line as “straight” at 0.4 degrees. Therefore, the M-CHARTS score is 0.4

The only treatment for ERM is the surgical removal of the ERM during vitrectomy to release the traction on the retina [16]. A previous prospective study showed that ERM and ILM peeling improved both best corrected visual acuity (BCVA) and metamorphopsia, as measured by the M-CHARTS (Inami). Specifically, at 12 months postoperatively, the BCVA improved from logMAR 0.33 ± 0.02 to 0.09 ± 0.02; the horizontal M-CHARTS score, from 1.05 ± 0.08 to 0.38 ± 0.06; and the vertical M-CHARTS score, from 0.89 ± 0.07 to 0.41 ± 0.06 [42]. However, clear criteria for surgical indications, which each ophthalmologist determines on the basis of the patient’s subjective symptoms and visual function test results, are lacking. Visual acuity is a useful indicator for evaluating visual dysfunction caused by ERM. However, relying solely on visual acuity to determine the indication for surgery is inappropriate as visual acuity often does not deteriorate in the early stages of ERM but is affected by cataracts. Therefore, metamorphopsia is more useful as an early symptom than visual acuity and is less affected by cataracts. Currently, the Amsler chart and M-CHARTS are commonly used methods for evaluating metamorphopsia (Fig. 2) [43, 44]. The Amsler chart is a 10-cm-square chart with grid lines, where patients are asked to indicate the distortion or waviness of the lines. This method is useful for screening for the presence or absence of metamorphopsia and for self-checking because it is simple and quick to perform. However, being a qualitative test, the Amsler chart cannot quantitatively evaluate the degree of metamorphopsia (Fig. 2a). Conversely, the M-CHARTS is an inspection sheet consisting of a straight line and 19 dotted lines with dot intervals ranging from 0.2 to 2.0 degrees of visual angle. The visual angle of the dotted line that is no longer perceived as distorted is defined as the M-CHARTS score (Fig. 2b). Thus, the M-CHARTS can quantitatively evaluate metamorphopsia, and the M-CHARTS score that interferes with daily life is approximately 0.5 [42, 44, 45]. Additionally, if the horizontal M-CHARTS score is < 0.9 preoperatively, the postoperative horizontal M-CHARTS value will be < 0.5, which may provide a reference for surgery indication acceptance [46].

In contrast to the M-CHARTS, a subjective test, retinal fold depth, an objective parameter, has been proposed as a criterion for ERM surgery [33]. In this report, the maximum fold depth measured using en face OCT images (maximum depth of retinal folds [MDRF]), which is proportional to the retinal traction force, was examined in relation to preoperative and postoperative M-CHARTS scores. The results suggested that the appropriate timing for ERM surgery is when the M-CHARTS scores are higher than 0.5 preoperatively and lower than 0.5 postoperatively; that is, when the MDRF in the parafoveal area is between 69 and 118 μm [33].

When the ERM and ILM are removed, retinal folds disappear the day after surgery [7]. By contrast, visual acuity and metamorphopsia improve slowly over several months after surgery but often do not improve completely [42]. ERM removal does not fully recover the amplitude of the oscillatory potentials in focal macular electroretinograms [47, 48]. The intraretinal cysts and ectopic inner foveal layers remain after ERM surgery [49–51]. Therefore, chronic inner retinal layer damage may be involved in the persistent metamorphopsia after ERM surgery.

### Controversy in ERM surgery

Consensus on whether to undertake ILM removal during ERM surgery has yet to be reached. The advantages of ILM peeling include (1) complete removal of the ERM and (2) reduction of postoperative ERM recurrence [52–55]. Regarding the preventive effect of ILM peeling on ERM recurrence, the rate of ERM recurrence was 2.0% in the ERM and ILM peeling group, as compared with 11.0% in the ERM peeling-only group [56]. However, many concerns about ILM peeling exist. For example, ILM peeling may be harmful to Müller cells because the ILM is the basement membrane. Dissociated optic nerve fiber layers are often observed after ILM peeling [57]; therefore, the retinal nerve fiber might also be damaged by ILM peeling. Furthermore, dyes for ILM staining could induce retinal toxicity. This is especially true for indocyanine green, regarding which several reports on retinal toxicity have been published, whereas brilliant blue G is considered relatively safe [58–62]. The impact of ILM peeling on visual acuity remains controversial, with conflicting meta-analyses reporting better outcomes with ERM peeling alone [63], no difference [56], or better outcomes with ERM and ILM peeling [64]. Additionally, patients with glaucoma have worse visual outcomes at 6 months and at the final follow-up after ERM surgery than those of patients without glaucoma [65]. Reports of decreased central sensitivity after ILM peeling in eyes with glaucoma also exist [66, 67]. Therefore, when performing ERM surgery, especially in patients with glaucoma, the surgeon should be careful to determine whether ILM peeling is required and to what extent the ILM is to be peeled.

**Fig. 3:**
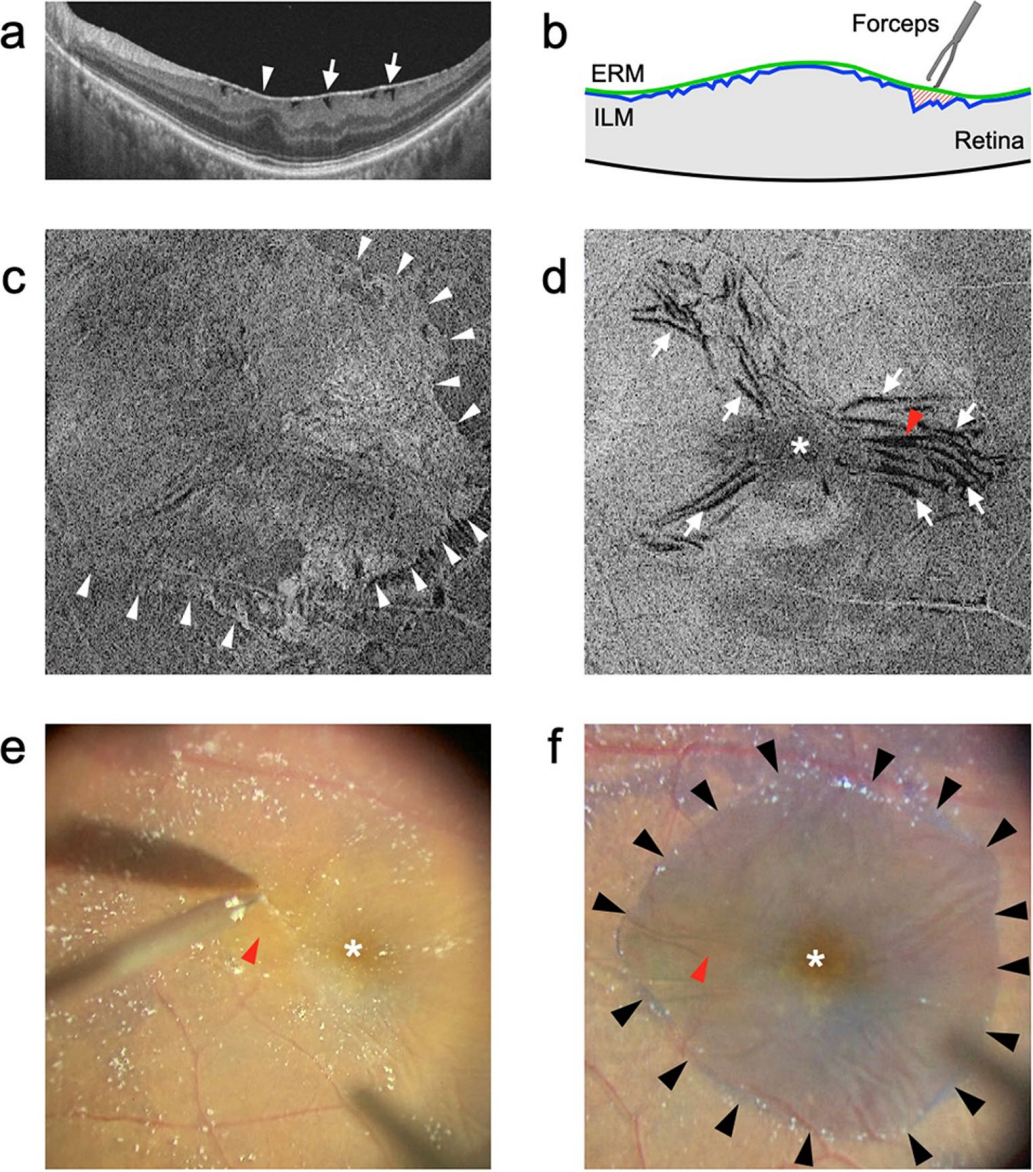
Representative case with epiretinal membrane (ERM) in which the patient underwent ERM removal without internal limiting membrane (ILM) peeling. **a** Horizontal B-scan optical coherence tomography (OCT) image, **c**,** d** en face OCT image flattened along the ILM, and **e**,** f** intraoperative images are shown. Asterisks in **d**, **e**, and **f** indicate the fovea. **a** ERM (arrowhead) with retinal folds (arrows) is observed. **b** Schematic image of the surgical technique. The ERM peeling is initiated over the wide ERM-ILM gap (red-shaded area) to grasp and peel only the ERM without damaging the ILM. **c** En face image at the ILM level demonstrates the ERM (arrowheads). **d** En face image 30 μm below the ILM level demonstrates the retinal folds (arrows). The widest fold (ERM-ILM gap; arrowhead) exists at the temporal side of the fovea. **e** The ERM peeling is initiated at the site with the widest ERM-ILM gap (arrowhead). **f** After the ERM has been peeled, the ILM is intact, as confirmed by blue staining with Brilliant Blue G, including the first grasping site (arrowhead)

Preoperative detailed OCT imaging may be important when attempting to remove only the ERM and to preserve the ILM to reduce the risk of adverse effects on glaucoma. A previous report revealed the usefulness of a surgical technique to visualize the spaces between the ERM and ILM gaps (ERM-ILM gaps) on en face OCT images and to avoid unexpected ILM peeling by initiation of membrane peeling at the site of the wide ERM-ILM gap (Fig. 3) [68]. Conversely, when peeling the ILM, keeping the peeled area to the minimum necessary may be one solution to minimize the adverse effects of ILM peeling. Retinal folds outside the parafoveal area (an area of a circle 3 mm in diameter centered on the fovea) did not affect visual acuity or metamorphopsia [7, 32]. Therefore, if only the ILM in the parafoveal area is peeled, the recurrence of ERM on the remaining ILM is unlikely to affect visual functions.

### Preretinal tissue resembling ERM: epiretinal proliferation

LMH was first reported as a macular morphology formed by the rupture of the inner wall of cystoid macular edema after cataract surgery [69]. After the advent of OCT, it became possible to observe cross sections of the macula in detail noninvasively, facilitating research on macular diseases, including LMH. Subsequently, many studies have revealed that the preretinal tissue in LMH differs from that of normal ERM, which was termed “dense ERM” or “atypical ERM” [70, 71]. In 2014, Pang and colleagues observed preretinal tissues in LMH in detail using spectral domain OCT and named them *lamellar hole-associated epiretinal proliferation* (LHEP) [72], and the term LHEP became widely used. Further studies revealed that LHEP is seen in LMH and other macular diseases [73]. This preretinal tissue is now referred to as *epiretinal proliferation* in the diagnostic criteria for LMH and related diseases based on OCT proposed by Hubschman and colleagues [74].

Epiretinal proliferation comprises a macular pigment-rich membranous tissue that surrounds the LMH. The presence of carotenoids in the epiretinal proliferation has been demonstrated by use of resonance Raman microscopy [75]. Epiretinal proliferation is observed as an isoreflective lesion at the foveal edge on B-scan images as well as a membranous structure on en face OCT images, usually without or with mild retinal folds [31, 34]. Histologic studies have shown that epiretinal proliferation shows high expression of glial fibrillary acidic protein, a glial cell marker, but low expression of α-smooth muscle actin [18, 70, 72]. This finding suggests that epiretinal proliferation is a membranous tissue composed mainly of Müller glial cells migrating from the inner retinal layers and lacking contractile properties, like traction on the retina. Understanding these characteristics and distinguishing between ERM and epiretinal proliferation is clinically crucial, particularly for determining the surgical approach (see the “Treatment of LMH, ERM foveoschisis, and MPH” section below).

### Diagnosis of LMH, ERM foveoschisis, and macular pseudohole

**Fig. 4:**
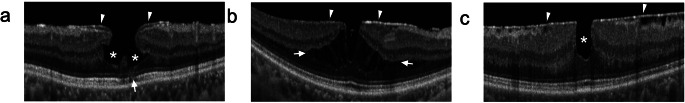
Representative cases of lamellar macular hole (LMH), epiretinal membrane (ERM) foveoschisis, and macular pseudohole (MPH). **a** A woman in her 60s with LMH. A B-scan optical coherence tomography (OCT) image demonstrates the irregular foveal structure with undermined edges (asterisks). Epiretinal proliferation (arrowheads) and ellipsoid zone disruption (arrow) are present. **b** A woman in her 60s with ERM foveoschisis. A B-scan OCT image demonstrates the presence of ERM (arrowheads) and foveoschisis (arrows). **c** A man in his 60s with MPH. A B-scan OCT image demonstrates the presence of ERM with retinal folds (arrowheads) and the verticalized foveal profile (asterisk)

The OCT-based diagnostic criteria by Hubschman and colleagues define the mandatory and optional criteria for the diagnosis of 3 diseases: LMH, ERM foveoschisis, and macular pseudohole (MPH) [74]. The mandatory criteria for LMH are (1) irregular foveal contour, (2) foveal cavity with undermined edges, and (3) at least 1 sign of evoking a loss of foveal tissue (thinning of the fovea at its center or around, and pseudo-operculum, which is a small opacity on the detached posterior hyaloid membrane). The optional criteria for LMH are (1) epiretinal proliferation; (2) foveal bump, which is an elevation of retinal tissue on the basis of the foveal cavity; and (3) ellipsoid line disruption. The mandatory criteria for ERM foveoschisis are (1) contractile ERM and (2) foveoschisis at the level of the Henle fiber layer, which is the foveal part of the outer plexiform layer, which is composed of bundles of Müller cells and photoreceptor axons. The optional criteria for ERM foveoschisis are (1) microcystoid spaces in the ILM, (2) retinal thickening, and (3) retinal wrinkling. The mandatory criteria for MPH are (1) foveal center-sparing ERM, (2) retinal thickening, and (3) verticalized or steepened foveal profile. The optional criteria for MPH are (1) microcystoid spaces in the INL and (2) near-normal central foveal thickness. Representative OCT images of LMH, ERM foveoschisis, and MPH are shown in Fig. 4.

An important consideration when diagnosing these 3 diseases is that their pathophysiologies often overlap. A detailed investigation of multiple OCT scans revealed that 34.1% of cases were “mixed types”; that is, different diagnostic criteria were satisfied in different OCT scans [76]. Therefore, a detailed examination of multiple OCT scans in different directions is necessary to accurately evaluate the pathophysiology of LMH and related diseases.

### Treatment of LMH, ERM foveoschisis, and MPH

**Fig. 5:**
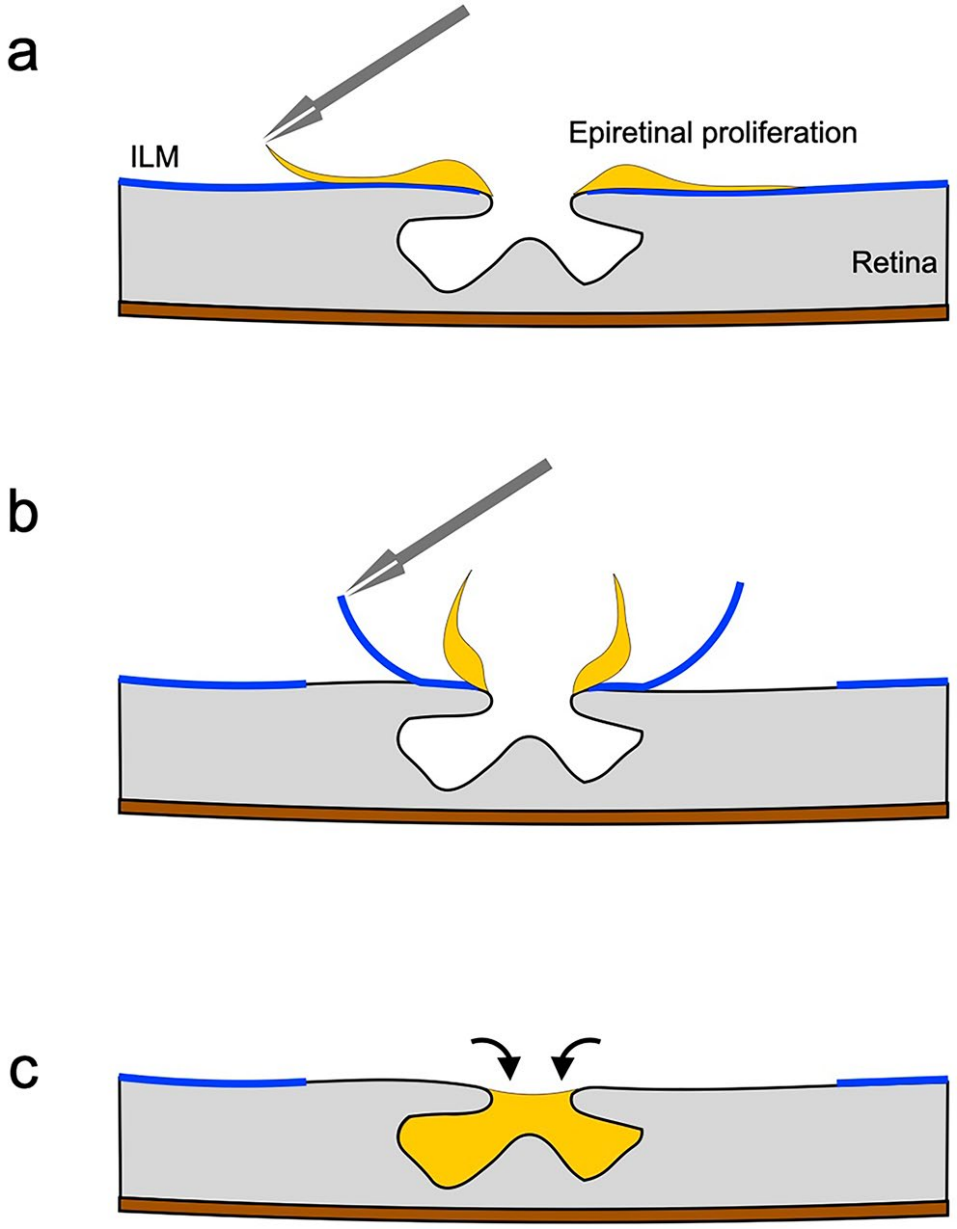
Schematic image of the epiretinal proliferation embedding technique for lamellar macular hole. **a** Lamellar macular hole with epiretinal proliferation. The forceps grasp the edge of the epiretinal proliferation. **b** The epiretinal proliferation is peeled; however, it is attached to the edge of the fovea. The internal limiting membrane (ILM) is grasped and peeled with the forceps. **c** The epiretinal proliferation is embedded into the foveal cavity (arrows). The ILM is peeled off

Established treatments for LMH are currently lacking; however, useful surgical procedures have been reported. Considering the pathophysiology, removal of the preretinal tissue is ineffective because LMH pathophysiology does not involve retinal traction. Additionally, a risk of postoperative full-thickness macular holes due to membrane removal exists because the fovea is often thin [77]. Therefore, a technique utilizing epiretinal proliferation, which is composed mainly of Müller cells, has been devised to embed epiretinal proliferation into the foveal cavity, and its usefulness has been demonstrated (Fig. 5) [78–82]. The ERM is a thin, semitranslucent membrane that adheres relatively strongly to the retina, whereas epiretinal proliferation is a yellowish-white, relatively thick tissue that can be easily detached from the ILM [83]. Utilizing the epiretinal proliferation to compensate for foveal tissue loss improves the foveal morphology and could restore visual function [78, 79, 81, 82]. Specifically, Takahashi and colleagues reported on 34 eyes with LMH that were treated with epiretinal proliferation embedding and subsequently followed for an average duration of 30.0 ± 17.7 months. They observed improvements in mean logMAR BCVA from 0.31 ± 0.25 to 0.10 ± 0.25 and in central retinal thickness from 123.2 ± 42.6 μm to 191.2 ± 42.6 μm [79]. Furthermore, just sparing epiretinal proliferation without embedding it has the same surgical result [84–86]. However, the evidence for the long-term outcome of epiretinal proliferation embedding or sparing surgery is insufficient, and further studies are needed to establish these techniques as the standard surgical treatment for LMH.

Moreover, for ERM foveoschisis and MPH, the only treatment is the surgical removal of the ERM, as both pathologies are retinal traction caused by ERM. The lines of evidence for these diseases are sufficient; hence, the surgical indications and visual prognosis can be similarly considered as ERM, as previously described [87–90]. Furthermore, each case should be evaluated from multiple perspectives, including visual acuity, metamorphopsia, and retinal traction, to determine the indication for surgery [91]. The decision whether to peel the ILM is also similar to that for ERM surgery. ILM peeling enables the complete removal of the ERM and reduction of postoperative ERM recurrence. However, potential problems with ILM peeling-induced damage to the Müller cells and retinal nerve fiber layer and retinal toxicity because of ILM staining dyes exist.

## Summary

**Fig. 6:**
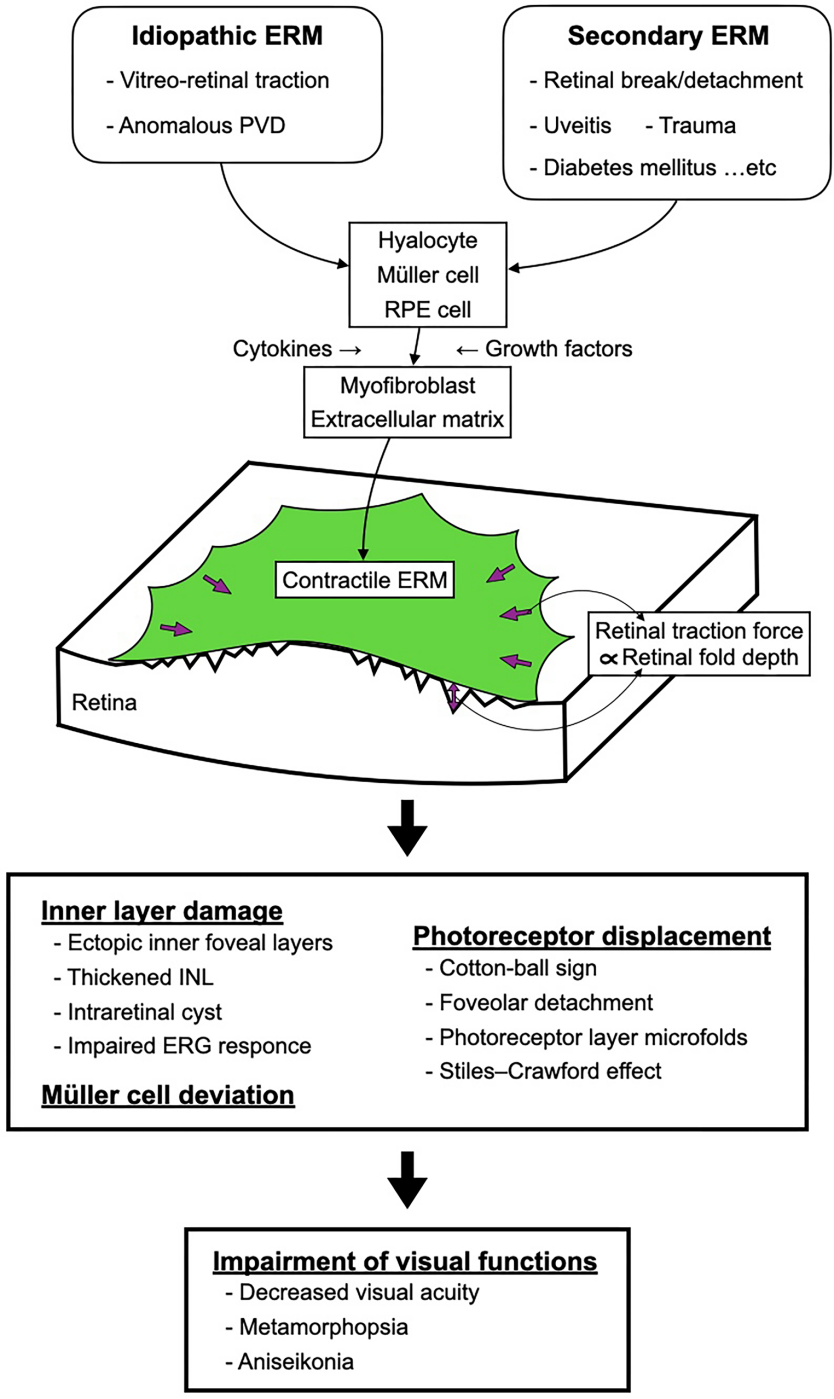
Schematic image of the pathophysiology of the epiretinal membrane (ERM). *PVD* posterior vitreous detachment, *RPE* retinal pigment epithelium, *INL* inner nuclear layer, *ERG* electroretinogram

This review has provided an overview and update on ERM, LMH, and related diseases. Figure 6 shows a schematic image summarizing ERM pathogenesis. Future studies should clarify the unresolved aspects of these diseases, including the detailed pathogenesis, surgical indications, and optimal surgical techniques.
